# *Treponema denticola*-Induced RASA4 Upregulation Mediates Cytoskeletal Dysfunction and MMP-2 Activity in Periodontal Fibroblasts

**DOI:** 10.3389/fcimb.2021.671968

**Published:** 2021-05-19

**Authors:** Erin Trent Malone, Sean Ganther, Nevina Mena, Allan Radaic, Keemia Shariati, Abigail Kindberg, Christian Tafolla, Pachiyappan Kamarajan, J. Christopher Fenno, Ling Zhan, Yvonne L. Kapila

**Affiliations:** ^1^ Kapila Laboratory, Department of Orofacial Sciences, School of Dentistry San Francisco, University of California San Francisco, San Francisco, CA, United States; ^2^ Bush Laboratory, Department of Cell and Tissue Biology, Biomedical Sciences Graduate, University of California San Francisco, San Francisco, CA, United States; ^3^ Fenno Laboratory, Department of Biological and Material Sciences & Prosthodontics, School of Dentistry, University of Michigan, Ann Arbor, MI, United States; ^4^ Zhan Laboratory, Department of Orofacial Sciences, School of Dentistry San Francisco, University of California San Francisco, San Francisco, CA, United States

**Keywords:** actin reorganization, matrix mettaloproteinase-2, RASA4, *Treponema denticola*, periodontal ligament (PDL)

## Abstract

The periodontal complex consists of the periodontal ligament (PDL), alveolar bone, and cementum, which work together to turn mechanical load into biological responses that are responsible for maintaining a homeostatic environment. However oral microbes, under conditions of dysbiosis, may challenge the actin dynamic properties of the PDL in the context of periodontal disease. To study this process, we examined host-microbial interactions in the context of the periodontium *via* molecular and functional cell assays and showed that human PDL cell interactions with *Treponema denticola* induce actin depolymerization through a novel actin reorganization signaling mechanism. This actin reorganization mechanism and loss of cell adhesion is a pathological response characterized by an initial upregulation of RASA4 mRNA expression resulting in an increase in matrix metalloproteinase-2 activity. This mechanism is specific to the *T. denticola* effector protein, dentilisin, thereby uncovering a novel effect for *Treponema denticola*-mediated RASA4 transcriptional activation and actin depolymerization in primary human PDL cells.

## Introduction

Periodontal disease is characterized by an altered periodontal ligament space, chronic inflammation, and destruction of the periodontal tissues, including a breakdown of the extracellular matrix (ECM) (Armitage, 2004). The periodontal complex consists of the periodontal ligament (PDL), alveolar bone, and cementum, which work together to turn mechanical load into biological responses that are responsible for maintaining a homeostatic environment (Jang et al., 2018). Cytopathic mechanisms in mechanotransduction are facilitated by cytoskeletal interaction with the ECM (Humphrey et al., 2014) and include ECM degradation and remodeling (Tsuji et al., 2004; Manokawinchoke et al., 2015), altered cellular differentiation (Kanzaki et al., 2002), altered cellular migration, and altered cellular adhesion or de-adhesion processes (Kang et al., 2010; Wang et al., 2019). A failure of mechanotransduction and actin organization within the periodontium results in loss of regeneration of periodontal tissues, remodeling of the ECM, and progression of disease; processes highly regulated within the PDL (Chukkapalli and Lele, 2018). The disease process is initiated by pathogenic microbes under conditions of microbial dysbiosis. *Treponema denticola*, an oral spirochete identified as a periodontal pathogen, is implicated in this dysbiosis leading to chronic inflammation, ECM remodeling, including enhanced MMP-2 activation, and periodontal tissue destruction (Takeuchi et al., 2001; Miao et al., 2011; Miao et al., 2014; Ruby et al., 2018).

Actin monomer and filament dynamics are necessary for mechanotransduction and are capable of regulating epigenetic enzymes (Sadhukhan et al., 2014; Serebryannyy et al., 2016), chromatin reprogramming (Zhao et al., 1998; Kapoor and Shen, 2014), transcriptional machinery (Visa and Percipalle, 2010), and gene expression (Misu et al., 2017). Mechanotransduction and actin dynamics can also influence homeostatic conditions towards disease through extracellular process and tissue destructive mechanisms (Humphrey et al., 2014). Actin dynamics are also involved in the regulation of tissue destructive genes, like matrix metalloproteinases (MMP) (Bildyug, 2016). MMP-2 is overexpressed in periodontal tissues compromised by apical periodontitis (Fernández et al., 2019), chronic apical abscesses (Letra et al., 2013), and chronic periodontitis (Ateia et al., 2018); implicating MMP-2 regulation in periodontitis. Major periodontopathogens, like *Porphyromonas gingivalis*, *Fusobacterium nucleatum*, and *Actinobacillus actinomycetemcomitans* can induce actin reorganization, thereby providing a possible mechanism through which microbial infection can facilitate periodontitis (Hassell et al., 1997; Yilmaz et al., 2003; Gutiérrez-Venegas et al., 2007; Hasegawa et al., 2008; Kinane et al., 2012; Zhang et al., 2013). *T. denticola* is also capable of affecting filamentous actin abundance in human gingival fibroblasts and epithelial cells (Baehni et al., 1992; Yang et al., 1998). Previous literature suggests that *T. denticola* induces subcortical actin polymerization through its effector protein, major surface protein (Wang et al., 2001; Amin et al., 2004; Visser et al., 2011). However, little is known regarding *T. denticola’s ability to* affect the actin dynamic function of human PDL cells (PDL) and the signaling components involved in the host response to *T. denticola* and effector protein dentilisin. In the present study, we characterize a novel mechanism demonstrating *T. denticola*-induced actin remodeling through stimulation of the Ca (2+)-dependent Ras GTPase-activating protein, RASA4 that further regulates MMP-2 activity. The overall objective of this study was to determine *T. denticola’s* influence on PDL cellular actin dynamics and the mechanism of action that may contribute to tissue destruction, including activation of MMP-2.

## Materials and Methods

### Human Subjects and IRB Approval

Institutional review board (IRB) approval for human subjects research was obtained *via* the University of California San Francisco institutional review board (# 16-20204; reference #227030).

### Periodontal Ligament Cell Harvesting and Cell Culture

Human primary periodontal ligament (PDL) cells were grown as explants from periodontal ligament tissues that were harvested from extracted teeth as previously described (Kapila et al., 1996). All cells were cultured in alpha minimal essential medium (αMEM) supplemented with 10% fetal bovine serum (Gibco, USA), 1% penicillin-streptomycin, and 1% amphotericin B (Gibco, USA). All cells were used between passages 3 and 7. Use of PDL cells for these studies was approved by the University of California San Francisco IRB. Two donor samples were pooled together per independent sample to eliminate variation for all experiments except shRNA experiments (only one donor per independent sample was used for these experiments).

### Microbial Culturing of *T. denticola*

*T. denticola* ATCC 35405 (American Type Culture Collection; Manassas, VA) and *T. denticola* mutants (CF522, isogenic dentilisin-deficient strain) (Godovikova et al., 2010), MHE (isogenic Msp-deficient strain) (Fenno et al., 1997), were cultured at 37°C under anaerobic conditions in oral treponeme enrichment broth (OTEB; Anaerobe Systems, Morgan Hill, CA). Culture purity was monitored using Syto 9 bacterial DNA dye and visualized in a fluorescent microscope for spirochete morphology. *T. denticola* was washed with αMEM cultured media, centrifuged, aspirated thrice, and finally resuspended in αMEM to be used for experiments. Bacteria used for all experiments never exceeded 6 passages.

### Purified Dentilisin

The dentilisin protease complex was purified from the detergent phase of the Triton X-114 extracts of *T. denticola* MHE by preparative sodium dodecyl sulfate-polyacrylamide gel electrophoresis (SDS-PAGE) using a model 491 Prep Cell (Bio-Rad Laboratories, Richmond, CA) as described previously (Fenno et al., 1998; Miao et al., 2011). Dentilisin purity was assayed in silver stained SDS-PAGE (Miao et al., 2011). Purified dentilisin migrated as a 100 kDa complex that upon heating resolves to its individual protein components: PrtP, PrcA1 and PrcA2, as documented previously (Miao et al., 2011).

### MOI Optimization

Multiplicity of infection (MOI) was optimized by performing a PDL cell viability assay with *T. denticola* at different MOIs (10, 50, and 100). This viability assay (Calcein AM; Thermo Fisher Scientific) was used according to manufacturer’s instructions. Cytotoxicity was encountered at an MOI of 500 (data not shown). In previous publications (Miao et al., 2014; Ateia et al., 2018), a *T. denticola* MOI of 50 rendered a strong cellular response in PDL cells without inducing cytotoxicity. Based on these observations, an MOI of 50 was considered optimal for studying the cellular response of PDL cells to *T. denticola* treatment.

### Treatment of PDL Cells With *T. denticola*

PDL cells were plated in 4-well chamber slides (Invitrogen), 8-well chamber slides (Invitrogen), or 60mm culture plates (Falcon) overnight at a density of 3x10^4^, 1.5x10^4^ or 1.0x10^6^ cells/well, respectively. Cells were then challenged with *T. denticola* at an optimized MOI of 50 for 2 h or left unchallenged as controls. Post-challenge, cells were washed three times with phosphate buffered saline (PBS, Gibco, USA) and incubated in αMEM media (Gibco, USA) supplemented with 10% fetal bovine serum (FBS; Gibco, USA), 1% amphotericin B (Gibco, USA), and 1% penicillin-streptomycin for 24 h.

### General Chemicals

All general chemicals were purchased from Sigma (St. Louis, MO, USA), unless otherwise noted.

### Imaging Stress Fibers *via* Immunofluorescence Staining

Human PDL cells were grown to confluency and passaged in sterile 4-well glass bottom plates (627870; Gibco, USA) 1.5x10^-5^ cells per well. Cells were initially challenged using the aforementioned protocol. Following incubation, cells were fixed with 4% paraformaldehyde for 15 mins at room temperature, washed with PBS, incubated with 0.2M glycine for 20 mins, washed with PBS, and permeabilized with 0.2% Triton X-100 for 2 mins. Then cells were washed with PBS, incubated with blocking buffer (10% fetal bovine serum in PBS) for 5 mins, washed with PBS, incubated with Hoechst nuclear probe (Sigma 33342; 1:3000) and SiR-Actin (Cytoskeleton Inc; probe for F-actin 1:5000) for 40 mins at 37°C, washed with PBS, and mounted with Faramount mounting media (Dako). All images were captured with a Leica TCS SP8 Confocal Laser Scanning Microscope built on a Leica DMi8 inverted confocal laser scanning microscope (NA 0.95, 506375, Leica). Leica Application Suite X (LASX) imaging software was used for image capture. Images were taken in the xy format at 63x or 20x magnification in oil immersion. LASX software preset fluorescence filters were used for the following: Hoechst (nuclear stain; ThermoFisher Scientific) and SiR-Actin Alexa Fluor 647 (F-actin stain; Cytoskeleton Inc.) The relative optical intensity (ROI) was measured using imaging analysis software (FIJI, Image J, National Institutes of Health).

### Live Imaging Immunofluorescence Staining Protocol

Human PDL cells (PDL) were grown to confluency and passaged in sterile 4-well glass bottom plates (627870; Gibco, USA) 1.5x10^-5^ cells per well. Before challenge, cells were incubated with SiR-Actin (1:5000 dilution) overnight, and Hoechst nuclear probe (Sigma 33342; 1:3000) for 30 mins. Additionally, wildtype (WT) *T. denticola* cells were incubated 20 mins before challenge with Syto 9 dye (1:500), vortexed and washed with αMEM media twice before challenge. Syto 9 staining has been used previously in bacterial culture assays with *T. denticola* and the stain did not interfere with the functioning or viability of the pathogen (Yamada et al., 2005). PDL cells were challenged using the aforementioned protocol. PDL cells treated with WT *T. denticola* (50 MOI) or control media were imaged at 15-minute intervals from 0 to 24 h at set landmarks. All images were captured with a Zeiss scanning confocal microscope at 20x magnification with fluorescence filters for Hoechst, Alexa Flour 488 filter (Syto 9 dye for DNA; ThermoFisher Scientific) and Alexa Fluor 647 filter (SiR-Actin probe). The relative optical intensity (ROI) was measured using imaging analysis software (FIJI, Image J, National Institutes of Health).

### Western Blot Analysis

PDL cells were plated in 60 mm tissue culture plates at 1.5x10^-6^ cells/plate overnight. Cells were then challenged with wildtype *T. denticola* at an MOI of 50 for 2 h or left unchallenged as controls. Post-challenge, cells were washed three times with PBS and incubated in αMEM medium for 24hrs. Cells were mechanically harvested with a cell-scraper, pelleted under centrifugation, washed with PBS, and lysed using radioimmunoprecipitation assay (RIPA) buffer. Lysates were then electrophoretically resolved on pre-made 4-12% bis-tris polyacrylamide gels (Invitrogen) and transferred onto immobilon-P PVDF transfer membranes (EMD Millipore) for western blotting analysis to evaluate changes in actin monomers (β-actin and γ-actin). Primary antibodies included: β-actin (Abcam; ab8227; RRID : AB_2305186), γ-actin (Santa Cruz; Cat# sc-65635, RRID : AB_1120816), and glyceraldehye 3-phosphate dehydrogenase (GAPDH) (Santa Cruz; sc-32233, RRID : AB_627679). Supersignal West Pico Plus Chemiluminescent substrate solution (Thermo Scientific) was used to develop protein bands. Densitometry was performed using FIJI imaging analysis software.

### RNA Sequencing

Three replicates of primary human periodontal ligament cells (PDL) were derived from three subjects. Cells were challenged with *T. denticola* treatment at a multiplicity of infection of 50 whereas control cells were challenged with αMEM media. At the end of the 24 h incubation period, samples were pelleted, frozen and sent to Novogene Corporation Inc. for RNA extraction, sequencing, and analysis. All samples passed quality control with a Q30 above 80% and sequencing was performed using the Illumina Platform PE150. The RNA-Sequencing data being presented is a subset of genes from the overall analysis. 165 genes were identified from the gene ontology pathways derived from the RNA sequencing data. Biological processes were identified from the top significant differentially regulated processes seen in the 5 h and 24 h gene ontology graphs (**Supporting Information, Figure 2** and **Figure 4**). Pathways identified were titled regulation of actin cytoskeleton organization, positive regulation of cell projection organization, regulation of actin filament organization, ras protein signal transduction, regulation of small GTPase meditated signal transduction, extracellular matrix component, and extracellular matrix organization. Based on these pathways, the top genes were identified and their adjusted p-value and differential expression was plotted as a volcano plot (**Figure 4**). All genes used in the volcano plot came from the 24 h challenge data sets within the pathways specified above. RASA4 was identified as one of the top genes upregulated upon the 24 h *T. denticola* challenge.

### Detachment Assay

Cells were challenged using the aforementioned protocol above. Following incubation, cells were vigorously washed with PBS three times. Vigorous washings included full force application of PBS with a 1mL pipette directly on cells and aspiration on high. This is different from normal culture methods; normally, PBS is added slowly on the side of the chamber/plate wall and aspirated on low. Cells were then fixed with 4% paraformaldehyde for 15 mins at room temperature, washed with PBS, incubated with 0.2M Glycine for 20 mins, washed with PBS, permeabilized with 0.2% Triton X-100 for 2 mins, washed with PBS, incubated with blocking buffer (10% fetal bovine serum in PBS; Gibco, USA) for 5 mins, washed with PBS (Gibco, USA), incubated with Hoechst (1:3000) and mounted with fluorescence mounting media (Faramount, Dako). Remaining cells were counted from 5 locations per well and cell number/location were averaged to constitute one experimental group. Mean cell count per experimental group was graphed as a ratio; control samples to wildtype *T. denticola* 50 MOI. Three samples were used per control and wildtype *T. denticola* groups. The images are from one representative experiment. The graph represents results from three separate experiments. Data were compared using a Student’s t-test. All images were captured with a Zeiss confocal microscope at 63x and 20x magnification with Hoechst and Alexa 647 (SiR-Actin) fluorescence filters, then cells were counted using imaging analysis software (FIJI).

### Gelatin Zymography

Gelatin zymography was conducted as previously described (Miao et al., 2011). Protein concentrations were calculated for each sample using the Pierce BSA Protein Assay kit according to manufacturer’s instructions (Cat # 23225, Thermo Scientific). Equal protein for each sample was mixed with 4x sample buffer (0.25 M Tris base, 0.8% SDS, 40% glycerol, and 0.05% bromophenol blue), loaded into each well, and subjected to SDS-PAGE at 4°C on 10% gels containing 2 mg/ml gelatin. After electrophoresis, SDS was removed from the gels by washing them in renaturing buffer (2.5% Triton X-100, 50 mM Tris base) twice for 30 min then gels were placed in developing buffer (50 mM Tris base, pH 8, 10 mM CaCl_2_, and 0.02% NaN_3_) for 30 mins. Developing buffer was changed and gels were incubated at 37°C for 16 h then stained with 0.05% Coomassie blue for 30 mins, and de-stained in 10% acetic acid and 40% methanol until clear bands in a blue background were visible.

### Quantitative RT-PCR (qRT-PCR)

RNA was isolated from PDL cells using the RNeasy Mini kit (Qiagen, Valencia, CA), reverse transcribed to cDNA using SuperScript Vilo cDNA Synthesis Kit (Invitrogen; Cat# 11754-050) and amplified by qRT-PCR using gene-specific primers for RASA4 (F: 5′-AGCGCAGCTCGCTGTACATC- 3’; R: 5′- GGCAGGTGCACTTGGTACTC- 3’), β-ACTIN (F: 5′-TGTTAGCGAGGGAGCAGTGG-3′; R: 5′-CCCATCGCCAAAACTCTTCA-3′), and GAPDH (F: 5′-TTGAGGTCAATGAAGGGGTC-3′; R: 5′-GAAGGTGAAGGTCGGAGTCA-3′) (Jin et al., 2007). Cycle threshold values of the genes of interest and the quantitative gene expression levels normalized to GAPDH for each experimental sample were determined and compared with that of unchallenged control samples.

### Transduction of PDL Cultures With RASA4 shRNA

Short hairpin RNA (shRNA) lentiviral particles specific for RASA4 (Santa Cruz Biotechnology; sc-89880-V) was used to inhibit RASA4 expression in PDL cells, according to manufacturer’s instructions. Briefly, PDL cells were incubated with polybrene reagent (EMD Millipore, USA) at a final concentration of 5ug/ml and RASA4 shRNA or scrambled shRNA (control) lentiviral particles (sc-108064) for 10 h. After infection, PDL cell media was changed with normal supplemented αMEM media overnight. Lentiviral particles used contain a puromycin resistance gene used for selection. Selection of clones with successfully intake of knockdown constructs were identified by treating infected hPDL cells with puromycin (Calbiochem, USA) at a final concentration of 5 µg/mL until single cell colonies remained. Experiment ended by washing with phosphate buffered saline (PBS, Gibco, USA) and replaced with normal growth medium (αMEM media supplemented with 10% fetal bovine serum (Gibco, USA), 1% amphotericin B (Gibco, USA), and 1% penicillin-streptomycin). Cells were passaged once and validated *via* RT-qPCR before used for experiments.

### Actin Dynamics Experiments

Actin polymerization for all experiments was stimulated by Jasplakinolide treatment (Jasp) of PDL cells at a concentration of 500nM for 1 h in αMEM alone media prior to *T. denticola* challenge and throughout *T. denticola* challenge as previously described. Control samples were challenged with αMEM alone media with no bacterial cells. These cells were labelled as Jasplakinolide alone. Actin depolymerization for all experiments was stimulated by Latrunculin B treatment (Lat B) of PDL cells at a concentration of 500nM for 1 h in αMEM alone media prior to *T. denticola* challenge and throughout *T. denticola* challenge as previously described. Control samples were challenged with αMEM alone media with no bacterial cells. These cells were labelled as Latrunculin B alone. Post-challenge, media was removed, washed with PBS thrice, and replaced with αMEM only media. Media was collected after the 24 h incubation for gelatin zymography. Cells were then incubated for 24h, then processed for RNA extraction for qRT-PCR.

### Statistical Analysis

All data used in this manuscript was analyzed using the statistical software GraphPad Prism version 8.4.3 (La Jolla California USA). Results were evaluated by The Student’s *t* test when comparing two groups. A one‐way analysis of variance (ANOVA) was used when comparing three or more groups. ANOVA Turkey’s multiple comparison test was used to distinguishing differences of two groups within experiments with three or more groups. The Two-way ANOVA test was used compare more than three groups, while comparing differences between shRNA scrambled and shRNA RASA4 knockdown samples. *P value of* ≤.05 was considered statistically significant. Annotation within figure legends identify *= p ≤.05, **= p ≤.01, ***= p ≤.001.

## Results

### PDL Cell Attachment and Contractility Is Impaired by *T. denticola* Challenge

**Figure 1:**
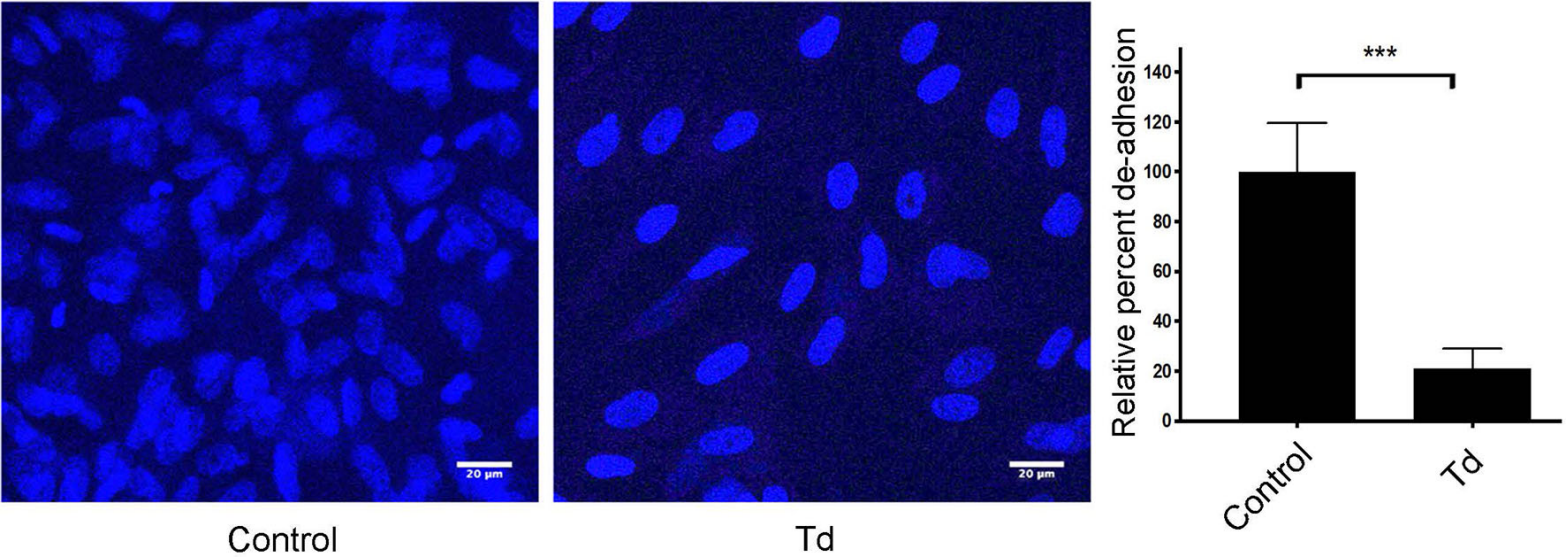
PDL cell attachment and contractility is impaired by *T. denticola* challenge. PDL cells were unchallenged (control) or challenged with *T. denticola* (50 MOI) for 24 h and processed for immunofluorescent staining with Hoechst (nucleus, blue), and imaged with Confocal Microscopy. Representative phase contrast images are shown, and graphs show the percent change in de-adhesion after 24 h. Data represent mean ± SD from three independent experiments. Data were compared using Student’s t-test. *** = p<0.001.

Since little is known about *T. denticola’s* effects on the actin filament processes of human PDL cells (PDL), we began this investigation by analyzing *T. denticola’s* modulation of cellular adhesion and live actin filament dynamics in PDL cells (**Figure 1** and **Supporting Information, Figure 1** video). *T. denticola* interactions with gingival fibroblasts disrupt cell adhesion (Baehni et al., 1992), therefore we examined *T. denticola’s* effects on PDL cell adhesion. *T. denticola* challenged PDL cells exhibited 70% more detachment than unchallenged cells (**Figure 1**). Live imaging of *T. denticola* challenged cells (**Supporting Information, Figure 1** and video) showed an impaired contractility leading to detachment of cells. Together, this indicates that *T. denticola* interaction with PDL cells negatively influences their actin dynamic properties. PDL cell interactions with *T. denticola* at an MOI of 50 were not cytotoxic to PDL cells as assessed with a Calcein AM viability assay (**Supporting Information, Figure 2**).

### *T. denticola* Decreases Stress Fiber and β-Actin Monomer Abundance in PDL Cells

**Figure 2:**
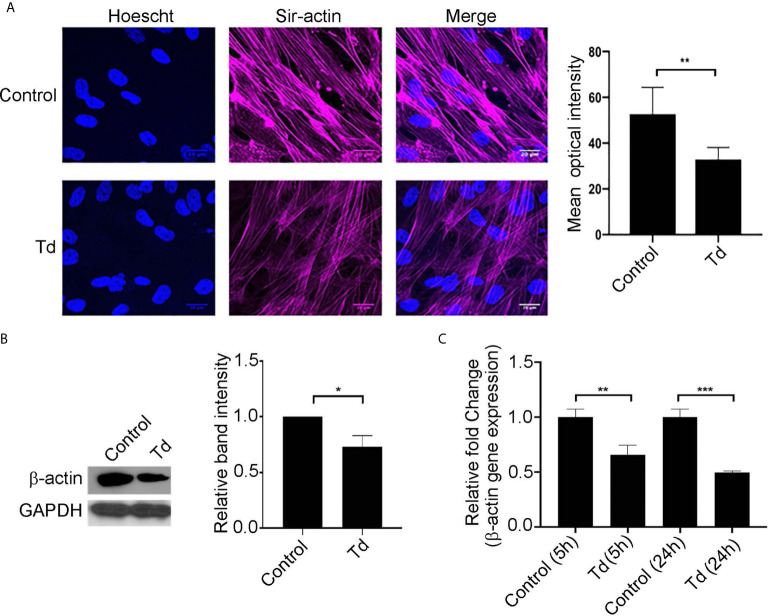
*T. denticola* challenge decreases stress fibers and β-actin protein monomer abundance in PDL cells. **(A)** (*Left*) PDL cells were unchallenged (control) or challenged with *T. denticola* (50 MOI) for 24 h and processed for immunofluorescent staining with Hoechst (nucleus, blue) and SiR-Actin probe, and imaged using confocal microscopy. (*Right*) Relative optical intensities were calculated from three independent experiments. **(B)** (*Left*) Representative immunoblot showing β-actin levels in PDL cells challenged with control medium or media containing *T. denticola* (50 MOI*)* for 24 h. (*Right*), Densitometry analysis of three independent experiments was performed using ImageJ software, and the ratio of control samples to *T. denticola* was calculated. Data represent mean ± SD from three independent experiments. Data were compared using Student’s t-test. * = p<0.05; ** = p<0.01. **(C)** β-actin gene expression in PDL cells challenged with control medium or media containing *T. denticola* (50 MOI) for 5h or 24 h by RNA-Seq. The graph shows the fold changes on a log2 scale. Data were compared using Student’s t-test. ** = p<0.01; *** = p<0.001.

Actin stress fibers are key to the mechanotransducing function of PDL cells (de Araujo et al., 2014). Therefore, we next analyzed *T. denticola’s* modulation of actin stress fibers and actin monomers in PDL cells (**Figure 2**). Treatment of PDL cells with *T. denticola* resulted in a significant decrease in stress fiber abundance as evaluated with immunofluorescence staining (**Figure 2A**). A 30% reduction in mean optical density was noted. To further evaluate *T. denticola’s* effect on actin fibers, β-actin protein expression was evaluated in this context. Western blotting analyses revealed that *T. denticola* reduced β-actin protein levels by approximately 30% in PDL cells (**Figure 2B**). RNA sequencing analyses further revealed that *T. denticola* also significantly decreased actin RNA levels in PDL cells at 5 and 24 hours by 60 and 76%, respectively (**Figure 2C**). Additionally, gene ontology enrichment analysis revealed a significant differential expression in the biological pathways related to actin and cytoskeletal organization within the 5 h challenge samples compared to the controls (**Supporting Information, Figure 3**).

### Gene Pathway and Volcano Plot Analyses Show RASA4 as a Top Upregulated Gene in *T. denticola* Challenged PDL Cells

**Figure 3:**
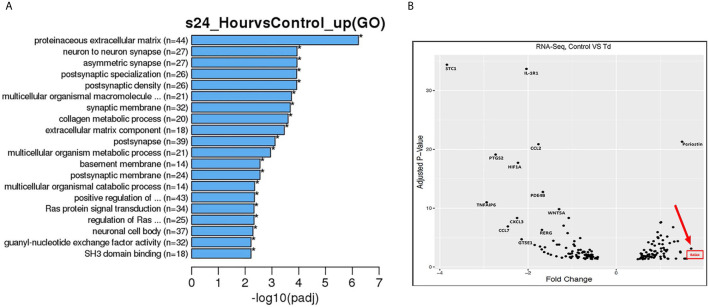
Gene pathways and volcano plot shows RASA4 as a top upregulated gene in *T. denticola* challenged PDL cells. PDL cells were unchallenged or challenged with *T. denticola* (50 MOI) for 24 h and differential gene expression analysis was performed using RNA-Seq. 165 genes were identified from the gene ontology pathways derived from the RNA sequencing data. Biological processes were identified from the top significant processes that were differentially regulated in the gene ontology enrichment analysis from 5 h and 24 h gene ontology graphs (**Supporting Information**, **Figure 3** and **A**). Pathways identified were titled regulation of actin filament organization, positive regulation of cell projection organization, regulation of actin filament organization, *ras* protein signal transduction, regulation of small GTPase meditated signal transduction, extracellular matrix component, and extracellular matrix organization. Based on these pathways, the top genes were identified and their adjusted p-value and differential expression was plotted as a volcano plot **(B)**. All genes used in the volcano plot came from the 24 h challenge data sets within the pathways specified above. RASA4 was identified as one of the top genes upregulated upon 24 h *T. denticola* challenge.

From the RNA sequencing data referenced previously, gene ontology (GO) enrichment data was derived for 5 h and 24 h challenged samples versus controls (**Supporting Information, Figure 3** and **Figure 3**). Among all biological pathways, actin and cytoskeletal regulatory pathways were significantly and differentially expressed at 5 h after *T. denticola* challenge. Actin cytoskeletal-related pathways were also among the top significantly and differentially expressed biological pathways, including the ras protein signal transduction and regulation of small GTPase mediated signal transduction pathways. In order to identify the top differentially expressed genes that were influenced by actin regulation and that could be upstream of MMP-2 regulation, we plotted genes that were identified within the significantly expressed biological processes from the gene ontology (**Figure 3A**). From these genes, RASA4 was identified as one of the top genes upregulated upon a 24 h *T. denticola* challenge. Since specific tissues are able to regulate MMP-2 activity *via* actin dynamics (Bildyug, 2016), we investigated the role of RASA4, as RASA4 is linked to actin reorganization and stress fiber maintenance. Interestingly, RASA4 was upregulated 1.7-fold in the *T. denticola*-challenged cells compared to controls (**Figure 3B**).

### Purified Dentilisin and *T. denticola* Upregulate RASA4 Gene Expression in PDL Cells

**Figure 4:**
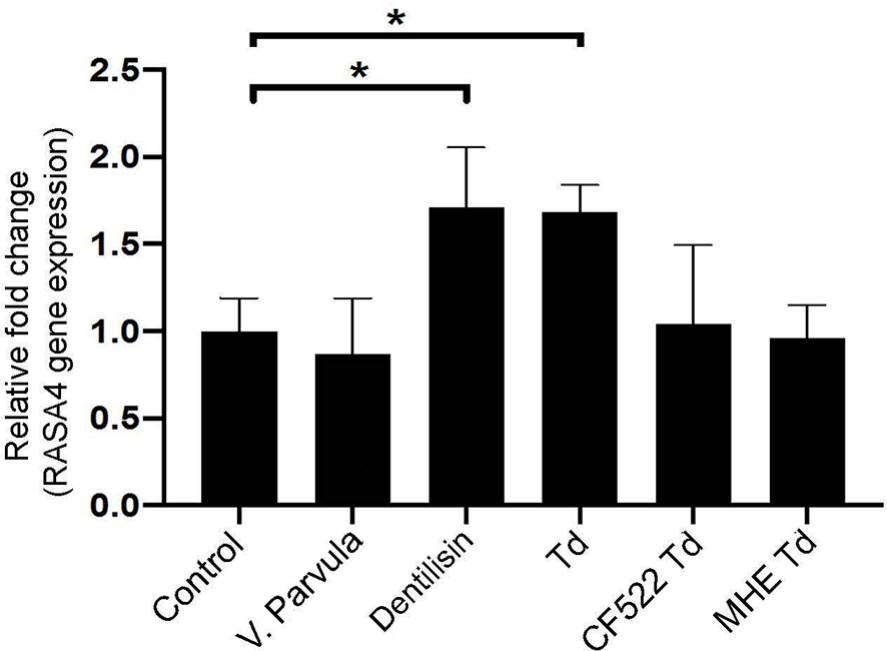
Purified dentilisin and *T. denticola* upregulate RASA4 gene expression in PDL cells. Quantitative RT-PCR analysis of RASA4 relative fold change in PDL cells challenged with *V. parvula*, wild type *T. denticola*, *T. denticola* dentilisin mutant CF522, *T. denticola* MHE or treated with purified dentilisin for 24 h. Data represent mean ± SD from three independent experiments. Data were compared using One-way ANOVA Tukey’s Multiple comparisons test. * = p<0.05.

Previous literature suggests that *T. denticola* induces subcortical actin polymerization through its effector protein, major surface protein (Msp) (Visser et al., 2011; Wang et al., 2019). Purified Msp can induce actin assembly by blocking the calcium influx into fibroblasts (Wang et al., 2001). Since RASA4 is activated by a calcium influx, the *T. denticola*-mediated mechanism of action in our system may be different. Therefore, we investigated the role of another *T. denticola* effector protein, dentilisin. We used purified dentilisin and a *T. denticola* isogenic mutant (CF522-Td; dentilisin null and Msp positive), which lacks the proteolytic PrtP subunit of dentilisin to determine if dentilisin was sufficient to enhance RASA4 upregulation. Additionally, we tested the *T. denticola* MHE strain (an isogenic Msp mutant that is dentilisin-positive) to investigate the role of Msp in this process. *Veillonella parvula* was used as an additional control, since it is a commensal Gram-negative bacterium. Quantitative RT-PCR results indicate that both purified dentilisin and wild type-*T. denticola* induced RASA4 expression (1.7 and 1.67-fold increase, respectively) (**Figure 4**). Neither CF522-Td, MHE-Td nor *V. parvula* had any effect on RASA4 expression compared to untreated PDL cells (**Figure 4**). Thus, our results indicate that while dentilisin contributes to *T. denticola*-mediated RASA4 transcriptional upregulation, Msp activity also contributes, presumably by a different mechanism.

### Jasplakinolide Inhibits *T. denticola*-Induced RASA4 Gene Expression, Whereas RASA4 Gene Expression Is Upregulated in Latrunculin B Pretreated and *T. denticola* Challenged PDL Cells

**Figure 5:**
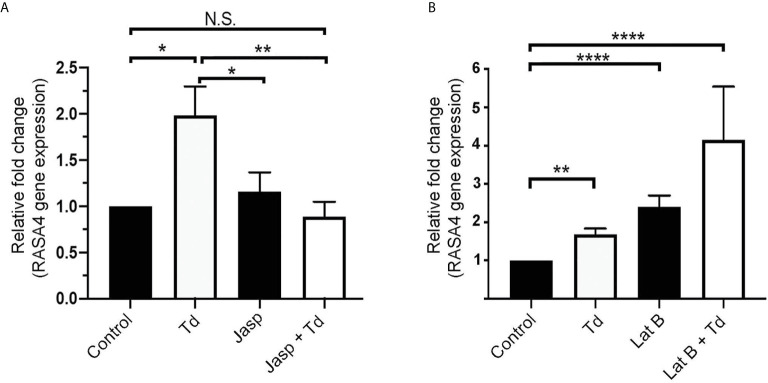
Jasplakinolide inhibited *T. denticola*-induced RASA4 gene expression, whereas RASA4 gene expression is upregulated in Latrunculin B pretreated and *T. denticola* challenged PDL cells. Quantitative RT-PCR analysis of RASA4 relative fold change in the gene expression of PDL cells treated with Jasplakinolide (Jasp; panel **A**) or Latrunculin B (Lat B; panel **B**) and challenged with *T. denticola* (50 MOI). Data represent mean ± SD from three independent experiments. Data were compared using Two-way ANOVA Tukey’s Multiple comparisons test. * = p<0.05; ** = p<0.01; **** = p<0.0001; N.S., not significant.

*T. denticola* causes actin reorganization in fibroblasts (Visser et al., 2011). In this study, we showed that *T. denticola* decreases actin stress fibers in PDL cells (F-actin intensity; **Figure 2**). Previous literature showed that RASA4 can disrupt actin stress fiber organization (Choi and Helfman, 2014) and induce actin reorganization (Choi and Helfman, 2014; Li et al., 2017). We hypothesized that RASA4 would also be upregulated during actin depolymerization but not during polymerization. Jasplakinolide (Jasp) or Latrunculin B (Lat B) were used to pre-treat PDL cells to induce actin polymerization or depolymerization, respectively. Post-treatment cells were collected for reverse transcriptase-quantitative PCR analysis. Results showed that Jasplakinolide did not have an effect on RASA4 gene expression compared to controls (**Figure 5A**). We next investigated the effect of actin depolymerization (which was induced by Latrunculin B pretreatment) on RASA4 gene expression. RASA4 gene expression was significantly upregulated (1.67 fold) with *T. denticola* and (2.4 fold) with Latrunculin B treatment compared to controls (**Figure 5B**). Combined treatment of Latrunculin B and *T. denticola* further upregulated RASA4 gene expression (4.15 fold). Thus, RASA4 upregulation is associated with *T. denticola*-mediated actin depolymerization and the actin depolymerization agent Latrunculin B.

### RASA4 Gene Expression Is Important for *T. denticola*-Mediated Actin Stress Fiber Dysfunction

**Figure 6:**
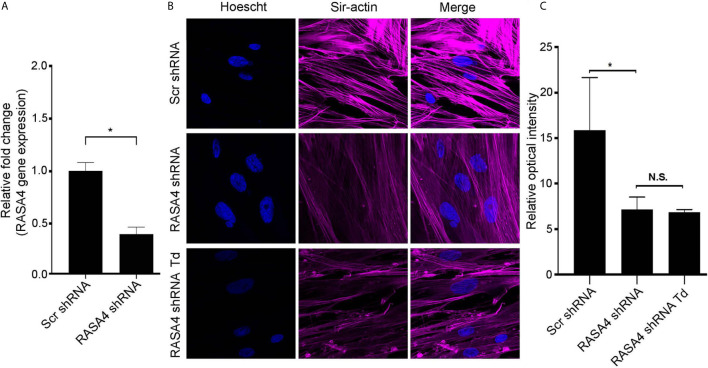
RASA4 gene expression is required for *T. denticola*-mediated actin stress fiber dysfunction. **(A)** Quantitative RT-PCR analysis of RASA4 relative fold change in PDL cells infected with scramble shRNA or RASA4 shRNA. **(B)** Representative confocal images and **(C)** relative optical intensity of stress fiber intensity in scramble shRNA, RASA4 shRNA and RASA4shRNA cells challenged cells with *T. denticola* (50 MOI) for 24 h and stained with SiR-Actin dye (1:5000). Data represent mean ± SD from three independent experiments. Data in panel **(A)** was analyzed using an Unpaired t test. Data in panel **(C)** was analyzed using One-way ANOVA Tukey’s Multiple comparisons test. * = p<0.05; N.S., Not significant.

To further determine the role of RASA4 in *T. denticola*-mediated actin depolymerization, we suppressed RASA4 gene expression in this context. RASA4 expression was reduced by 60% in hPDL cells infected with RASA4 shRNA lentiviral particles compared to control infected cells (**Figure 6A**). These cells were then challenged with or without *T. denticola*. After challenge, cells were stained with actin to visualize cellular stress fiber abundance by confocal microscopy. PDL cells infected with shRNA RASA4 exhibited reduced stress fiber abundance and organization compared to control cells treated with scrambled shRNA (Scr shRNA) (**Figure 6B**). Image analysis of actin stress fiber staining intensity showed that shRNA RASA4 infected cells exhibited a 2.4-fold decrease compared to control infected cells (**Figure 6C**). In contrast, *T. denticola* challenge did not change the levels of actin staining intensity any further. These data indicate that RASA4 gene expression is important for homeostatic actin organization and for *T. denticola*-mediated depolymerization of stress fibers in PDL cells.

### Actin Depolymerization Through *T. denticola* Challenge and Latrunculin B Pretreatment Increases MMP-2 Enzymatic Activity

**Figure 7:**
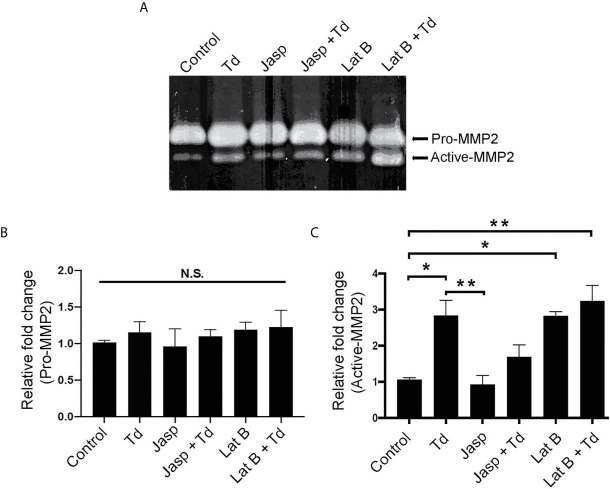
Actin depolymerization through *T. denticola* challenge and Latrunculin B pretreatment increases MMP-2 enzymatic activity. Actin polymerizing agent Jasplakinolide (Jasp) and actin depolymerizing agent Latrunculin B (Lat B) were used to pretreat PDL cells for one h. Cells were then unchallenged or challenged with *T. denticola* (50 MOI) for 24 h. Cultured media was collected and gelatin zymography was used to measure MMP-2 activity. A representative Gelatin zymogram is shown **(A)** and densitometry analysis of pro-MMP-2 **(B)** and active-MMP-2 **(C)**. Data represent mean ± SD from three independent experiments. Data were compared using One-way ANOVA Tukey’s Multiple comparisons test. *. = p<0.05; ** = p<0.01; N.S., not significant.

Actin dynamics have been shown to regulate MMPs (Bildyug, 2016). Additionally, we previously showed that MMP-2 activation increased upon *T. denticola* challenge (Miao et al., 2014). Thus, we investigated whether *T. denticola*-mediated changes in actin dynamics could modulate MMP-2 activity in PDL cells and if the actin-depolymerizing effect of the chemical inhibitor Latrunculin B (Lat B) would affect MMP expression in this context. As in **Figure 5**, Latrunculin B (Lat B) or Jasplakinolide (Jasp) were used to pre-treat PDL cells to induce actin depolymerization or polymerization, respectively. Following pre-treatment, *T. denticola* challenged PDL cells were collected for gelatin zymography to analyze MMP-2 activity (**Figure 7A**). There were no significant changes in the inactive form of MMP-2 (pro-MMP-2) among any of the samples (**Figure 7B**). Induction of actin polymerization with Jasplakinolide did not result in any difference in MMP-2 activation compared to control (**Figure 7C**). Challenging PDL cells with *T. denticola* alone significantly increased MMP-2 activity 2.8-fold, but pre-treatment with Jasplakinolide before *T. denticola* challenge decreased MMP-2 activity (1.6 fold compared to control), thereby abrogating the effect of *T. denticola* on MMP-2 (**Figure 7C**). In contrast, induction of actin depolymerization in PDL cells with Latrunculin B alone showed a notable increase in active MMP-2 bands (2.8-fold) compared to control (**Figure 7C**). Additionally, samples pretreated with Latrunculin B, then challenged with *T. denticola*, also increased MMP-2 activity (3.2 fold) compared to control. These data indicate that actin dynamics regulate MMP-2 activity, and *T. denticola* mediates these MMP-2 effects through an actin depolymerization mechanism.

### RASA4 Gene Expression Is Required for *T. denticola*-Mediated Enhancement of MMP-2 Activity

**Figure 8:**
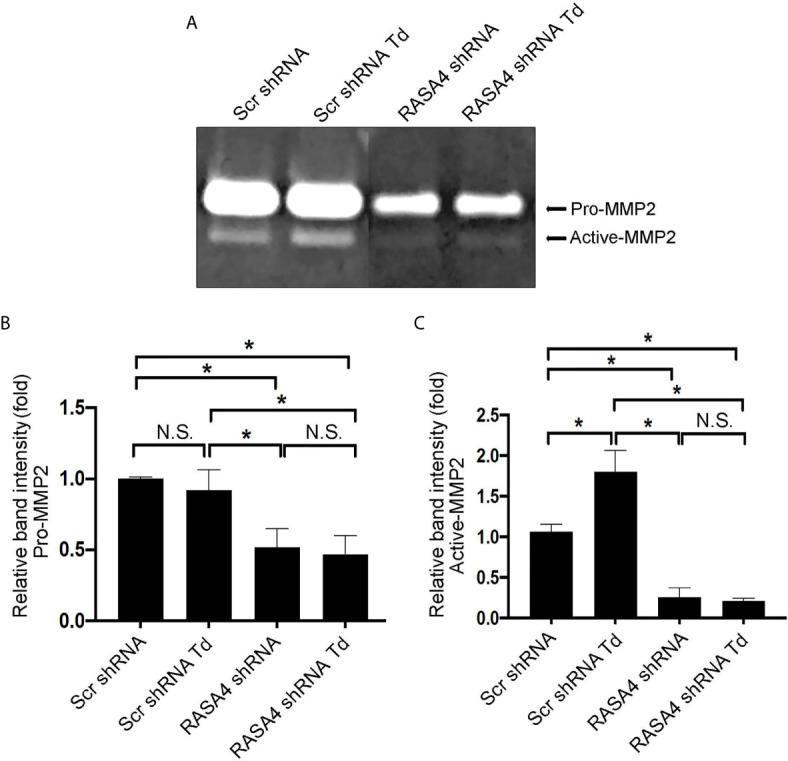
RASA4 gene expression is required for *T. denticola*-mediated enhancement of MMP-2 activity. Pro-MMP-2 and active-MMP-2 in scramble shRNA and RASA4shRNA cells challenged with *T. denticola* (50 MOI) for 24 h. **(A)** a representative zymogram. **(B)** (Pro-Mmp-2) and **(C)** (active Mmp-2) show mean ± SD from three independent experiments. Data was compared using Two-way ANOVA Tukey’s Multiple comparisons test. * = p<0.05; N.S., not significant.

Since RASA4 is required for *T. denticola*-mediated actin depolymerization and since actin dynamics mediate changes in MMP, we next investigated whether RASA4 is required for *T. denticola*-mediated changes in MMP-2 activity in PDL cells. Using the same experimental set up as in **Figure 6**, we obtained culture media from each group to analyze MMP-2 activation. Gelatin zymography of culture media from these cells showed that *T. denticola* challenge significantly increased active MMP-2 levels (2.5 fold) in controls (Scr shRNA cells plus *T. denticola)* compared to unchallenged (Scr shRNA) cells (**Figures 8A–C**). In contrast, shRNA RASA4 infected cells exhibited decreased MMP-2 activity (1.67-fold) compared to controls (Scr shRNA), and *T. denticola* was unable to increase the levels of MMP-2 activity in the context of RASA4 shRNA (**Figures 8A–C**). This indicates that RASA4 is required for *T. denticola* driven induction of depolymerization and subsequent regulation of MMP-2 activity in PDL cells.

## Discussion

In this study, we show that in *T. denticola*-PDL cell interactions*, T. denticola* drives enhanced MMP-2 activity through a novel actin reorganization signaling mechanism. This reorganization mechanism is a pathological response characterized by upregulation of RASA4, depolymerization of actin filaments, and a subsequent increase in MMP-2 activity. This mechanism is specific to the *T. denticola* effector protein dentilisin (although Msp is also important), thereby uncovering a novel effect for *T. denticola*-mediated actin depolymerization in primary human PDL cells that is distinct from that previously reported for *T. denticola* Msp in human gingival fibroblasts. Although there are different mechanisms reported for *T. denticola*’s contribution to actin reorganization, Jobin et al. found that *T. denticola* purified Msp induces Ca(2+) entry and actin reorganization in cultured fibroblasts (Jobin et al., 2007). This is contrary to other findings by Ko et al. who reported that outer membrane extracts of *T. denticola* inhibited calcium influxes in gingival fibroblasts and disrupted actin-dependent processes (Ko et al., 1998). This suggests that the bacterial source (purified versus bacterial extracts) is important in determining host effects on actin organization and calcium influxes, but knowledge of the overall effects of the whole bacterium is lacking. Furthermore, using *T. denticola* mutant strains carrying defined defects in proteins of interest to assay their biological relevance is important. Earlier literature showed that Latrunculin B treatment induced calcium influxes though that study did not assay effects of a defined Msp mutant strain (Wang et al., 2001). We show here that the calcium influx-triggered gene, RASA4, is upregulated in the presence of Latrunculin B and its expression is further increased in the presence of *T. denticola*. We also show the importance of *T. denticola* dentilisin and the Msp proteins in this process. While neither the dentilisin mutant nor the Msp mutant showed effects on RASA4 expression significantly different from controls, further studies are required to determine whether a double mutant would show a distinguishable difference. Taken in aggregate, these data indicate that dentilisin and Msp are key *T. denticola* proteins important in regulating actin assembly and calcium influx through RASA4 is mechanistically important (Ko et al., 1998; Jobin et al., 2007; Visser et al., 2011).

There are various signaling pathways implicated in actin organization and stress fiber formation. *T. denticola*-mediated actin reorganization is stimulated by PIP-2 dependent signaling leading to activation of the small GTPases RAC1, RhoA, and Ras in fibroblasts (Visser et al., 2011). To further evaluate the global effects of *T. denticola* on the PDL cytoskeleton at the mRNA level, we performed RNA sequencing of PDL challenged cells. RNA sequencing analysis of *T. denticola*-challenged PDL cells compared to non-challenged cells revealed a significant differential expression of genes related to actin cytoskeletal dynamics, including the actin monomer (β-actin), γ-actin, IQGAP3, ACTR3, ACTR2, ARPC3, ARPC5, RERG, and ARHGEF4. Gene ontology also confirmed that Ras signaling was amongst the top 20 most upregulated pathways. When examining actin organization and *ras* signaling genes, a candidate gene RASA4 was identified as a key upregulated gene (increased 1.7-fold, p-value 7.62E-04) in *T. denticola* challenged cells. Its paralog RASA4B was also upregulated 1.57-fold (p-value 3.82E-02). We confirmed that *T. denticola* and its secreted protease dentilisin are responsible for RASA4 gene upregulation. This is consistent with previous reports identifying RASA4 as important for microbial immune responses to bacterial infection (Zhang et al., 2005). Our study suggests that RASA4 may be a key cellular response to periodontal infection and a possible biomarker for *Treponema denticola* and *Treponema denticola*-associated diseases.

RASA4 can constitutively interact with Rac1 and Cdc42, and it inhibits the RAS/ERK signaling pathway, which is important for maintaining actin organization. Inhibition of the RAS/ERK pathway *via* RASA4 disrupts actin stress fiber organization (Choi and Helfman, 2014) and induces actin reorganization (Choi and Helfman, 2014; Li et al., 2017). This evidence suggests that RASA4 may be a key regulator of actin stress fiber reorganization stimulated by *T. denticola* challenge. By knocking down RASA4 and showing no difference in F-acin abundance with or without *T. denticola*, our results show that RASA4 is required for actin dynamics, specifically, actin depolymerization in the presence of *T. denticola.* Our data is consistent with other reports showing that actin reorganization is induced by *T. denticola.* Other publications suggest that this is facilitated by the effector protein Msp (Visser et al., 2011). Given the current data with the mutant *T. denticola* strains highlighting the role of dentilisin and the importance of the Msp, it is possible that RASA4 is acting through a unique mechanism as a result of both dentilisin and the Msp.

RASA4 has not been previously linked to MMP-2 activity. Our results demonstrate that a periodontal pathogen, *T. denticola* relies on RASA4 genetic expression to induce actin stress fiber disruption and increase MMP-2 activity in PDL cells. In order to confirm that RASA4 was involved in enhanced MMP-2 activity, a suppression strategy was used. Successful suppression of RASA4 expression effectively decreased overall pro-MMP-2 activity and active MMP-2. In the presence of *T. denticola*, MMP-2 activity is normally enhanced in PDL cells (Miao et al., 2011). Our study suggests that this increase is due to actin depolymerization of actin stress fibers, but suppression of RASA4 abrogated the effect of *T. denticola* on these actin components and MMP-2. These results indicate that RASA4 mRNA expression is required for actin-mediated enhanced MMP-2 activity. This study is the first to characterize RASA4 as a key player in *T. denticola-*mediated actin reorganization and MMP-2 activity in PDL cells.

Several periodontal pathogens have been reported to increase MMP-2 expression and activity. Known red and orange complex bacteria (Socransky et al., 1998), *Porphymonas gingivalis*, *Actinobacillus actinomycetemcomitans*, and *Fusobacteria nucleatum* are able to stimulate MMP-2 production and secretion in human epithelial cells and PDL cells (Pattamapun et al., 2003; Tiranathanagul et al., 2004; Gursoy et al., 2008). We have previously shown that *T. denticola* increases both activation of MMP-2 proteolytic activity and transcription through a chronic, epigenetic mechanism (Miao et al., 2011; Ateia et al., 2018), resulting in MMP-2-dependent fibronectin fragmentation (Miao et al., 2011).

Additionally, activators of MMP-2 are affected by *Treponema denticola* interactions. Tissue inhibitors of metalloproteinases, specifically TIMP-1 and -2, are important in the inhibition and activation of MMP-2. Previous studies have shown that *T. denticola* challenge of PDL cells upregulates TIMP-2 and MMP-14 mRNA expression (Miao et al., 2014). Dentilisin is important in the increase in protein levels of TIMP-2 and MMP-14. (Miao et al., 2014) Furthermore, purified dentilisin can breakdown TIMP-1 and TIMP-2 (Nieminen et al., 2018). These conditions would favor a ratio for maximum MMP-2 activation. Together, these studies suggest that bacterial stimulation of MMP-2 and its co-activators/modulators by periodontal pathogens plays a role in tissue destruction; however, the mechanism(s) involved for individual species and host cell types are not well understood. The present study examined RASA4 gene expression as a regulator for *T. denticola*-mediated enhancement of MMP-2 activity. Calcium-dependent signaling can also induce MMP-2 expression in multiple cell types, including human periodontal fibroblasts and oral squamous cell carcinoma cells (Munshi et al., 2002; Osorio et al., 2015). Consistent with this finding, we show that RASA4 (which relies on calcium signaling) is necessary for *T. denticola* interaction to increase MMP-2 activity. Additionally, our results indicate that *T. denticola*-mediated actin reorganization requires RASA4 gene expression. Actin reorganization regulates MMPs in different ways; showing tissue specificity (Bildyug, 2016). MMP-2 expression can increase or decrease due to actin dynamics; polymerization and depolymerization (Sanka et al., 2007; Bildyug, 2016). For example, inhibition of actin polymerization had no effect on MMP-2 expression in HT1080 fibrosarcoma cells, whereas in human trabecular meshwork cells, inhibition of actin polymerization induced the activation of MMP-2 expression (Chintala et al., 1998; Sanka et al., 2007). We examined whether actin dynamics are involved in our system and found that sequestering actin monomers (which depolymerizes actin stress fibers) by treatment with latrunculin B, increased active MMP-2 activity in PDL cells.

MMP-2 and MMP-9 are the major gelatinases amongst MMPs. *T. denticola* is associated with MMP-9 in gingival crevicular fluid from periodontally challenged patients compared to healthy patients (Yakob et al., 2013). PMN interaction with Treponeme components, chiefly the 53-kDa protein and LPS, increased the release of MMP-9 (Ding et al., 1996). The MSP effector protein, enhances the production of MMP-9 in peripheral blood monocytes (Gaibani et al., 2010). In isolation, purified dentilisin does have the ability to convert purified proMMP-9 to active MMP-9 (Nieminen et al., 2018). MMP-9 may play a role in the context of this paper, but PDL cells do not make large amounts of MMP-9. PDL cells are major producers of MMP-2. Clinically, this is relevant because MMP-2 plays a key role in fibronectin cleavage. The presence of 40, 68, and 120kDa fibronectin (FN) fragments in gingival crevicular fluid are markers of periodontal disease (Huynh et al., 2002). PDL cells create these FN fragments predominately through enhanced production of MMP-2 activity and mRNA expression in response to *T. denticola* and purified dentilisin challenge (Huynh et al., 2002; Miao et al., 2011; Miao et al., 2014). Knockdown of MMP-2 nearly eliminates FN fragments (Gaibani et al., 2010). Therefore MMP-2 was the relevant MMP in this study, but further study may elucidate MMP-9 playing a significant role (Miao et al., 2011; Yakob et al., 2013).

In summary, the current study highlights that *T. denticola* mediates actin reorganization (depolymerization) through RASA4 gene expression, which enhances MMP-2 activity in human PDL cells. RASA4 was identified as a novel contributor to the PDL host response and tissue destruction process. The study also identifies that actin dynamics play a role in the progression of periodontal disease, specifically actin depolymerization as a key contributor to enhance MMP-2 activity. These contributions shed light on new potential targets for *T. denticola*-PDL interactions that influence the progression of periodontitis and the maintenance of a healthy periodontium.

## Data Availability Statement

The data presented in the study are deposited in the FigShare repository, ascension number 10.6084/m9.figshare.14336909 (**Supplementary Figure 1** High Resolution Videos) and 10.6084/m9.figshare.13530566 (RNA sequencing processed data).

## Ethics Statement

The studies involving human participants were reviewed and approved by Institutional review board (IRB) approval for human subjects research was obtained *via* the University of California San Francisco institutional review board (# 16-20204; reference #227030). Written informed consent for participation was not required for this study in accordance with the national legislation and the institutional requirements

## Author Contributions

EM: Investigation, Project Administration, Methodology, Formal Analysis, Funding Acquisition, Writing (original draft, review and editing), Supervision Validation, and Visualization. SG: Data curation, Methodology, and Investigation. AR: Conceptualization, Methodology, and Supervision. KS: Investigation. NM: Investigation and Validation. CT: Investigation. AK: Methodology, Investigation, and Resources. PK: Project Administration, Supervision, and Writing (original draft, review and editing). JF: Conceptualization, Resources, Funding Acquisition, Supervision, and Writing (original draft, review and editing). LZ: Resources. YK: Conceptualization, Resources, Supervision, Funding Acquisition, and Writing (original draft, review and editing). All authors contributed to the article and approved the submitted version.

## Funding

This work was supported by funding from NIH (R01 DE025225 to YK and JF; F30 DE027598 to EM).

## Conflict of Interest

The authors declare that the research was conducted in the absence of any commercial or financial relationships that could be construed as a potential conflict of interest.

## Supplementary Material

Supplementary Figure 1
*T. denticola* interaction negatively influences PDL cell actin filament functioning. Approximately 1.0x10^5 PDL cells per well were plated in a 4-well glass bottom culture plate. SiR-Actin dye (1:5000) and Hoechst (1:3000) stain were added to each well 4 hours before imaging. *T. denticola* was pre-incubated in a 15mL falcon tube with Syto9 (1:500) fluorescent dye for 20 mins. Bacteria were washed and centrifuged twice to remove unbound Syto9 and cells were reconstituted with αMEM media. Syto9-labelled *T. denticola* (50 MOI) was added to plated PDL cells. Control cells contained no *T. denticola.* Culture plates were imaged in a sterile chamber containing 5% CO2 and connected to a scanning laser confocal microscope. Images were set to image every 15 minutes for 24 hours. Maximum projections (representative image) and each individual image were analyzed in FIJI software. Hoechst, SiR-Actin, and Syto9 were assigned blue, cyan, and green filters, respectively. Images were combined to create the imaging video. Link to high resolution videos are under the data availability statement.

Supplementary Figure 2
*T. denticola* interaction does not affect viability of PDL Cells. PDL cells were plated in 96 well plate (approximately 3x 10^4^ cell per well) unchallenged (control) or challenged with *T. denticola* (10, 50, or 100 MOI) for 24 h and Calcein AM assays were performed according to manufacturer’s instructions. Data represent mean ± SD from three independent experiments containing 10 wells per sample. Data was compared using One-way ANOVA Tukey’s Multiple comparisons test. *= p<0.05; N.S.= not significant

Supplementary Figure 3
*T. denticola* interaction reflects an effect on actin and cytoskeletal biological processes. Gene ontology enrichment was performed from RNAseq data previously described. Gene ontology for PDL cells challenged for 5 h with *T. denticola* versus control (unchallenged PDL cells) showed a significant differential expression. Top differentially expressed biological processes include: regulation of actin filament organization and actin filament-based process, actin filament organization, regulation of cytoskeletal organization, regulation of actin organization, extracellular matrix organization.
