# Supplementary figures and images for: Treponema denticola-Induced RASA4 Upregulation Mediates Cytoskeletal Dysfunction and MMP-2 Activity in Periodontal Fibroblasts
Source: Front Cell Infect Microbiol. 2021 May 19;11:671968. doi: 10.3389/fcimb.2021.671968 (PMC8171266; doi:10.3389/fcimb.2021.671968)

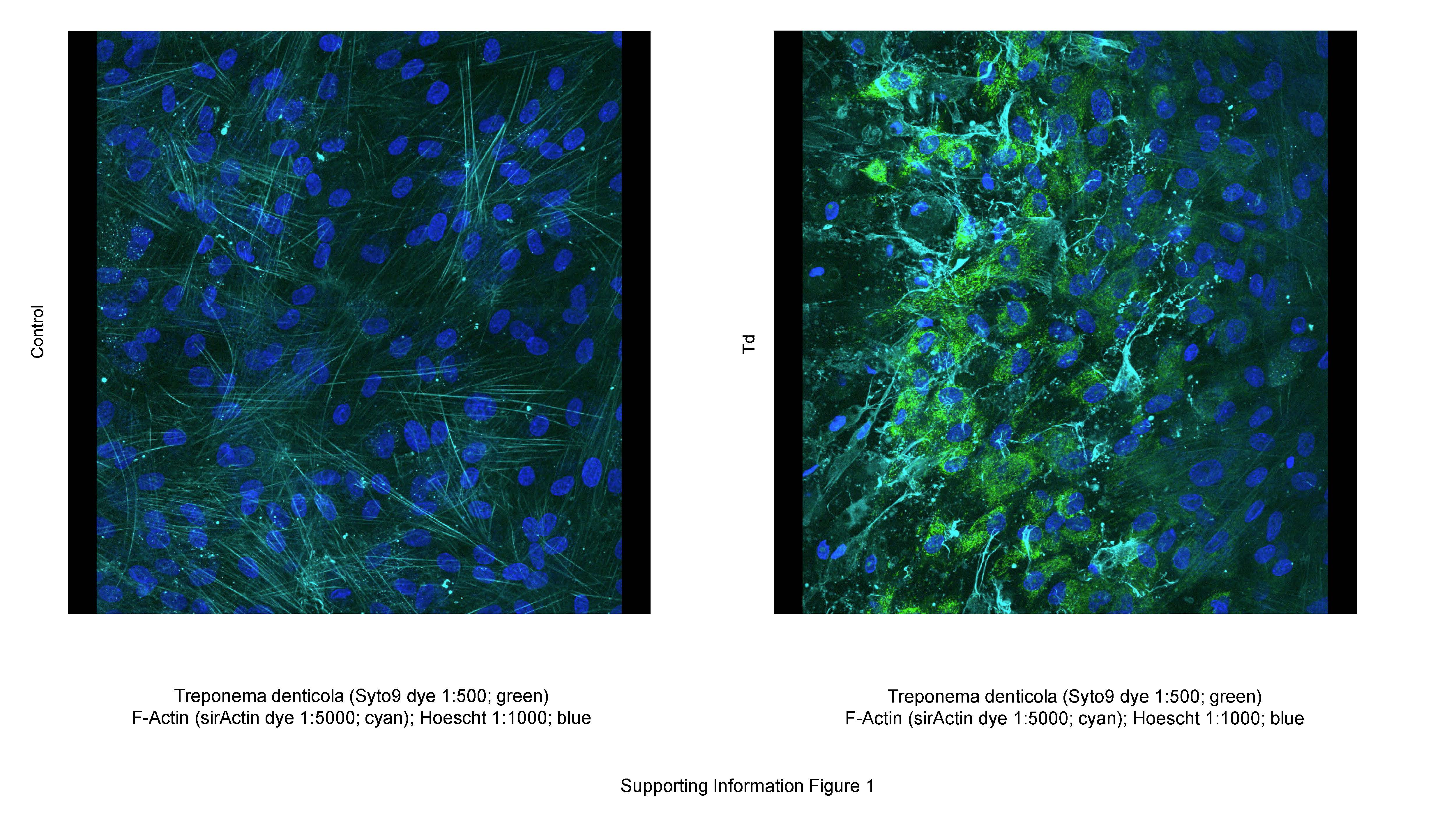

Supplement: Supplementary Figure 1 — T. denticola interaction negatively influences PDL cell actin filament functioning. Approximately 1.0x10^5 PDL cells per well were plated in a 4-well glass bottom culture plate. SiR-Actin dye (1:5000) and Hoechst (1:3000) stain were added to each well 4 hours before imaging. T. denticola was pre-incubated in a 15mL falcon tube with Syto9 (1:500) fluorescent dye for 20 mins. Bacteria were washed and centrifuged twice to remove unbound Syto9 and cells were reconstituted with αMEM media. Syto9-labelled T. denticola (50 MOI) was added to plated PDL cells. Control cells contained no T. denticola. Culture plates were imaged in a sterile chamber containing 5% CO2 and connected to a scanning laser confocal microscope. Images were set to image every 15 minutes for 24 hours. Maximum projections (representative image) and each individual image were analyzed in FIJI software. Hoechst, SiR-Actin, and Syto9 were assigned blue, cyan, and green filters, respectively. Images were combined to create the imaging video. Link to high resolution videos are under the data availability statement. [file Image_1.jpeg]

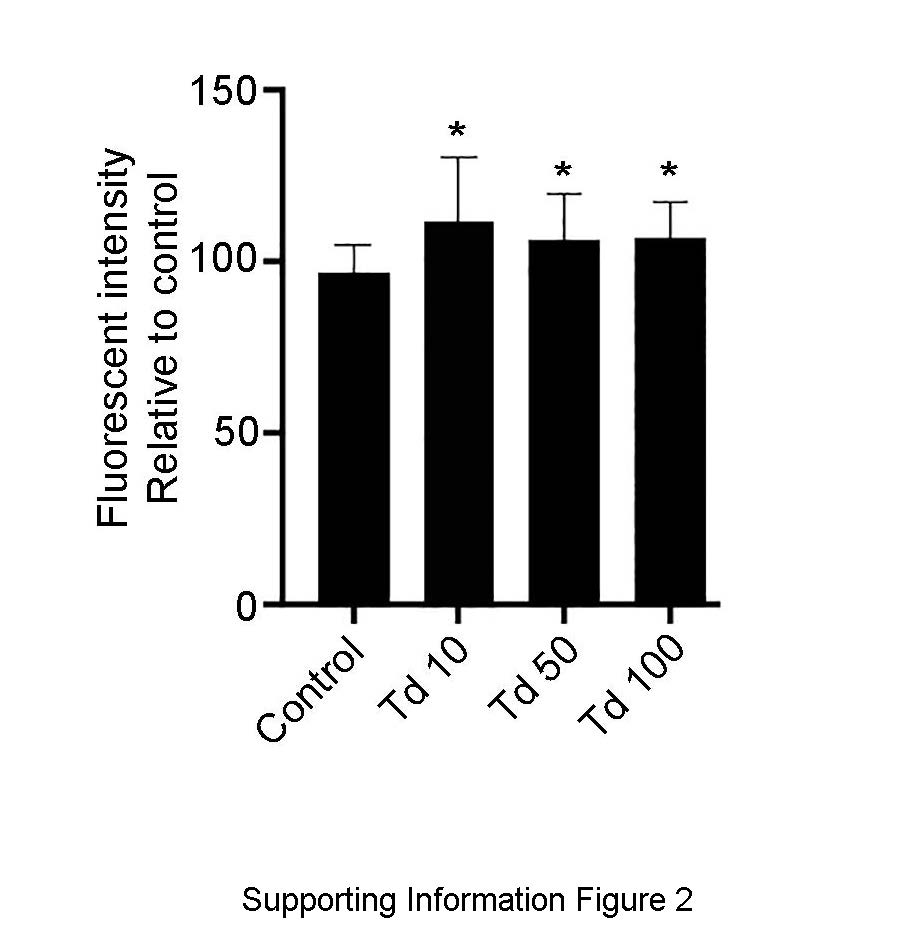

Supplement: Supplementary Figure 2 — T. denticola interaction does not affect viability of PDL Cells. PDL cells were plated in 96 well plate (approximately 3x 10^4^ cell per well) unchallenged (control) or challenged with T. denticola (10, 50, or 100 MOI) for 24 h and Calcein AM assays were performed according to manufacturer’s instructions. Data represent mean ± SD from three independent experiments containing 10 wells per sample. Data was compared using One-way ANOVA Tukey’s Multiple comparisons test. *= p<0.05; N.S.= not significant [file Image_2.jpeg]

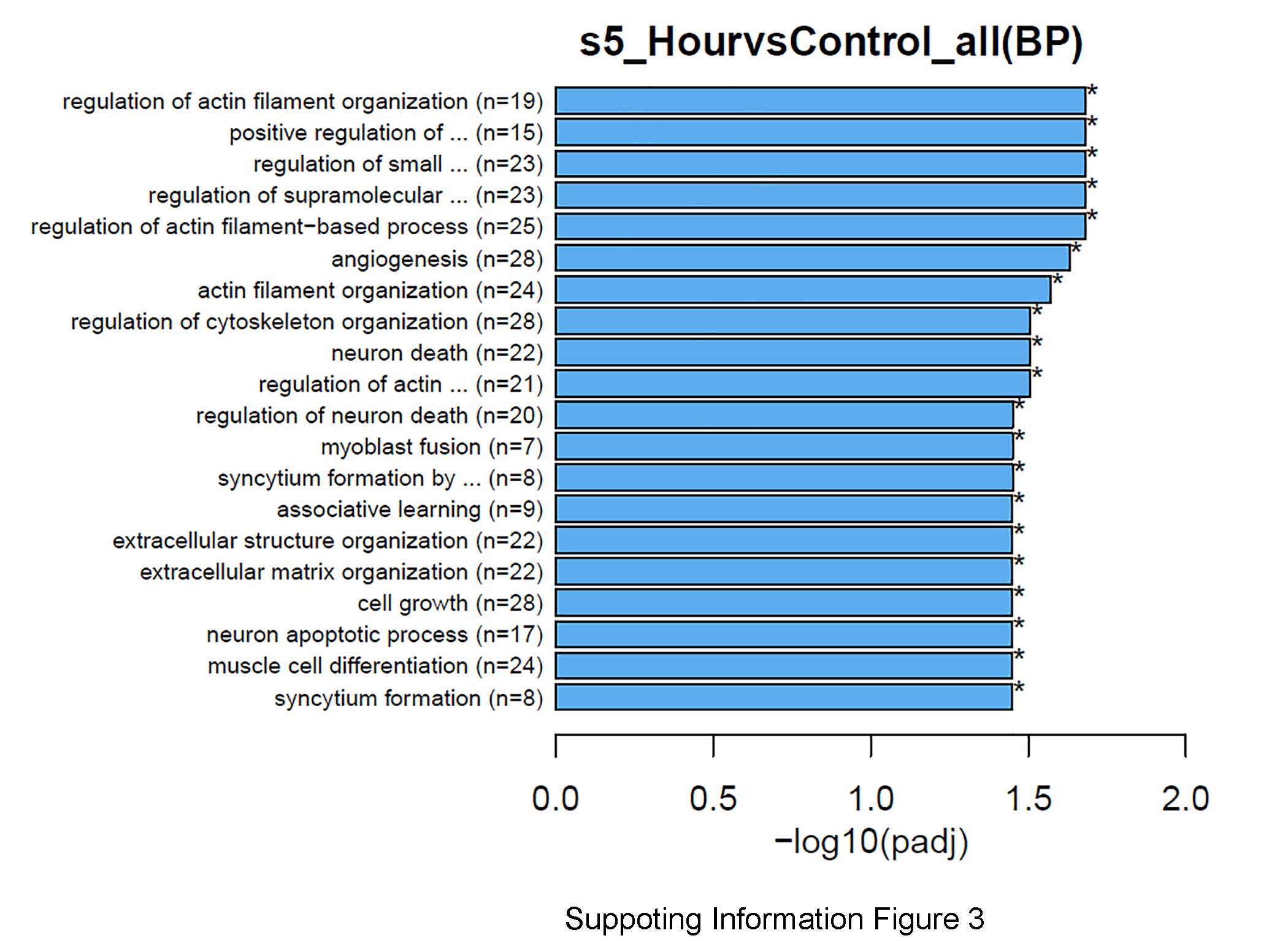

Supplement: Supplementary Figure 3 — T. denticola interaction reflects an effect on actin and cytoskeletal biological processes. Gene ontology enrichment was performed from RNAseq data previously described. Gene ontology for PDL cells challenged for 5 h with T. denticola versus control (unchallenged PDL cells) showed a significant differential expression. Top differentially expressed biological processes include: regulation of actin filament organization and actin filament-based process, actin filament organization, regulation of cytoskeletal organization, regulation of actin organization, extracellular matrix organization. [file Image_3.jpeg]
